# Syphilitic Aortitis Incidentally Detected by Fluorine-18 Fluorodeoxyglucose (18F-FDG) PET/CT

**DOI:** 10.7759/cureus.94353

**Published:** 2025-10-11

**Authors:** Kazunori Seo, Hiroshi Oiwa

**Affiliations:** 1 General Medicine, Hiroshima City Hiroshima Citizens Hospital, Hiroshima, JPN; 2 Emergency Medicine, Hiroshima City Hiroshima Citizens Hospital, Hiroshima, JPN; 3 Rheumatology, Hiroshima City Hiroshima Citizens Hospital, Hiroshima, JPN

**Keywords:** 18f-fdg–pet/ct, aortitis, syphilis, tertiary syphilis, treponema pallidum

## Abstract

Syphilis shows a variety of symptoms at various stages; it is a sexually transmitted infection caused by *Treponema pallidum*. Syphilitic aortitis is a recognized complication of tertiary syphilis that may progress to aortic aneurysm formation. Because this condition can be fatal, early diagnosis is crucial to optimize patient outcomes. We describe a rare case of syphilitic aortitis incidentally detected by Fluorine-18 fluorodeoxyglucose (18F-FDG) PET/CT. Utilization of 18F-FDG PET/CT imaging can facilitate the identification of syphilitic aortitis during its subclinical phase, thereby enabling timely intervention and potentially averting severe or fatal complications.

## Introduction

Syphilis, caused by *Treponema pallidum*, shows a range of symptoms throughout its different stages. Uncomplicated syphilitic aortitis can occur in 70-80% of untreated cases, with complications including aortic regurgitation, coronary ostial stenosis, and aortic aneurysms occurring in 10%; aneurysms may form over 15-30 years [1,2], though in the era of effective antibiotic therapy, syphilitic aortitis has become an exceptionally rare clinical entity [3]. After the introduction of penicillin, the incidence of syphilis across all stages in the United States decreased by approximately 95% between 1943 and 2000 [4], but aortitis caused by syphilis can be fatal and should therefore be diagnosed at an early stage. This case presentation refers to the importance of considering syphilis in the differential diagnosis of aortitis even in the absence of typical late-stage manifestations and demonstrates the utility of imaging modalities in monitoring treatment response.

## Case presentation

**Figure 1:**
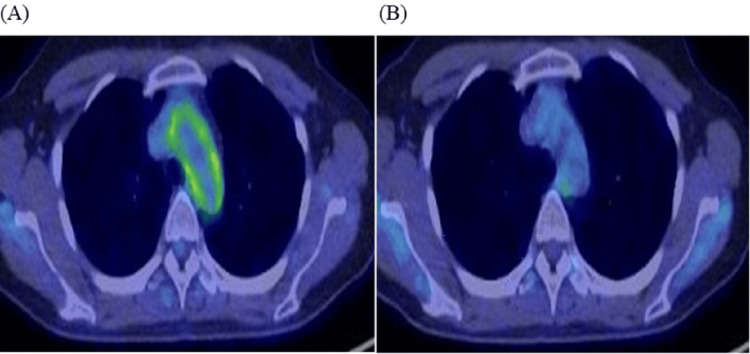
18F-FDG PET/CT scan findings A: showed increased metabolic activity in the ascending aortic wall; B: after six months of treatment, the 18F-FDG PET/CT scan showed that aortic arch uptake had disappeared. 18F-FDG: Fluorine-18 fluorodeoxyglucose

A 35-year-old woman underwent a regular Fluorine-18 fluorodeoxyglucose (18F-FDG) PET/CT scan after surgery for breast cancer. The 18F-FDG PET/CT scan performed one month prior to presenting at our institution showed increased metabolic activity (SUV max 3.8) in the ascending aortic wall (Figure 1A). She had no symptoms, including fever, malaria, or loss of body weight, and no symptoms suggestive of cardiovascular involvement, such as chest pain, dyspnea, or palpitations. Blood examinations showed a slight leukopenia and C-reactive protein (CRP) level of 0.5 mg/dL, and the erythrocyte sedimentation rate (ESR) is also elevated at 47 mm/hour. Based on the findings of 18F-FDG PET/CT, we suspected the possibility of Takayasu's disease. However, serological tests for syphilis were positive, with rapid plasma reagin (RPR) at 2000 R.U. and *Treponema pallidum* hemagglutination assay (TPHA) at 125562 T.U. (Table 1), suggesting active syphilitic vasculitis. HIV antibody screening was performed and found to be non-reactive. She was treated for one month with amoxicillin and probenecid. After six months of treatment, uptake of the aortic arch on 18F-FDG PET/CT had disappeared (Figure 1B), and one year later, RPR quantification had decreased to 287 R.U. Finally, we diagnosed her as having syphilitic aortitis.

**Table 1:** Blood test and serologic test results for syphilis and HIV CRP: C-reactive protein; ESR: erythrocyte sedimentation rate; RPR: rapid plasma reagin; TPHA: *Treponema pallidum *hemagglutination assay

Test Name	Result	Reference Range
Leukocytes	3.2 × 10³/µL	4.0–11.0 × 10³/µL
Hemoglobin	11.7 g/dL	12-16 g/dL
Platelets	219 × 10³/µL	150–450 × 10³/µL
CRP	0.5 mg/dL	0-0.14 mg/dL
ESR	47 mm	0-15 mm
TPHA	125562 T.U.	0-9.99 T.U.
RPR	2000 R.U.	0-0.99 R.U.
HIV antibody test	Negative	Negative

## Discussion

Syphilis, caused by *Treponema pallidum*, shows a variety of symptoms at various stages. Syphilitic aortitis is a known complication of tertiary syphilis and can cause aneurysm of the aorta [5]. Although this condition has been rare due to the widespread use of antimicrobial agents, aortitis caused by syphilis can be fatal and should therefore be diagnosed at an early stage [3]. The differential diagnosis of aortitis encompasses both infectious etiologies, most commonly involving *Salmonella, Staphylococcus*, or *Streptococcus* species, and non-infectious conditions, such as vasculitis of the great vessels (including giant cell arteritis and Takayasu arteritis), IgG4-related disease, and aortitis secondary to systemic inflammatory disorders [6]. It is important to include syphilis in the differential diagnosis when aortitis is observed. Several case reports have described accumulation observed in the aortic arch by 18F-FDG PET/CT, leading to a diagnosis of syphilitic aortitis, with some cases undergoing 18F-FDG PET/CT for treatment assessment; however, such cases remain exceedingly rare [5,7-10]. It is important to include syphilis in the differential diagnosis when aortitis is observed. In our literature search, several cases of syphilitic arteritis diagnosed by PET/CT have been reported to date [5,7-11], and to our knowledge, this is the first documented case in which the condition was incidentally detected in an asymptomatic, non-HIV-infected patient. Performing an 18F-FDG PET/CT scan may allow the early diagnosis of syphilitic aortitis at a subclinical stage and prevent life-threatening or fatal outcomes [7,11].

## Conclusions

This case presentation refers to the importance of considering syphilis in the differential diagnosis of aortitis even in the absence of typical late-stage manifestations. It demonstrates the utility of imaging modalities in monitoring treatment response. Therefore, the sensitivity, specificity, and practical application of 18F-FDG PET/CT scans in both the diagnosis and monitoring of syphilitic aortitis require further evaluation.
